# Prospective Long-Term Follow-Up of Pulmonary Diffusion Capacity Reduction Caused by Dose-Dense Chemotherapy in Patients with Breast Cancer

**DOI:** 10.1155/2019/2584859

**Published:** 2019-10-28

**Authors:** Yosef Landman, Salomon Marcello Stemmer, Aaron Sulkes, Victoria Neiman, Tal Granot, Daniel Hendler, Mordechai Reuven Kramer, Karen Gelmon, Rinat Yerushalmi

**Affiliations:** ^1^Institute of Oncology, Davidoff Cancer Center, Rabin Medical Center, Petach Tikva, Israel; ^2^Sackler School of Medicine, Tel Aviv University, Tel Aviv, Israel; ^3^Institute of Pulmonology, Rabin Medical Center, Petach Tikva, Israel; ^4^Department of Medical Oncology, British Columbia Cancer Agency, Vancouver, Canada

## Abstract

**Background:**

Our previous study of pulmonary function in 34 patients with early breast cancer without preexisting lung disease showed that anthracycline- and taxane-based adjuvant dose-dense chemotherapy (DDC) caused a significant 16.4% mean reduction in carbon monoxide diffusing capacity (DLCO). The present study reports the pulmonary and oncological outcomes of these patients on long-term follow-up.

**Patients and methods:**

The primary endpoint was DLCO measured by the pulmonary function test (PFT) performed at a median of 27 months after DDC (range, 8–97) in 25 patients without disease recurrence. DLCO values were recorded as a percentage of predicted values according to age, height, and hemoglobin level and analyzed relative to baseline pre-DDC DLCO values. The secondary endpoints were symptoms, additional therapies, and cancer outcomes during a median of 11 years' follow-up (range, 4.4–11.4).

**Results:**

A longitudinal general linear model showed significant effects of time on DLCO and its trend (*F*(1, 87) = 14.68, *p* < 0.001 and *F*(1, 87) = 10.26, *p*=0.002, respectively). Complementary descriptive analysis showed a significant recovery on the follow-up PFT (75.6% vs. 81.9%, *p*=0.002), but it was still significantly lower than the baseline DLCO (81.9% vs. 92.0%, *p*=0.003). Five patients (20%) still showed a >20% relative DLCO reduction from baseline. Patients with dyspnea or fatigue at later clinical follow-up had a significantly lower DLCO value on the follow-up PFT than nonsymptomatic patients (80.5% vs. 92.1%, *p*=0.02). DLCO recovery was inversely correlated with age (*R* = −0.39, *p*=0.05), but no significant correlation was found with the length of time until the follow-up PFT or additional therapies. There was no association of DDC-related DLCO reduction with cancer outcomes.

**Conclusions:**

The significant reduction in DLCO seen after DDC in patients with potentially curable breast cancer is evident years afterwards, especially in older patients. While most patients partly recover, some will have a lasting symptomatic DLCO impairment.

## 1. Introduction

Better therapies and screening techniques for patients with breast cancer along with the general aging of the population have increased the number of breast cancer survivors [1–3]. This has made late toxicities from cancer care an ever-growing problem [2–4]. Awareness of the potential dangers of therapy is mandated for all practitioners and patients, as the adverse effects on daily function and health-related quality of life (HRQOL) can last many years. While dyspnea and acute pulmonary toxicity during breast cancer care have been extensively reported and studied [5–9], data on its long-term pulmonary sequelae remain sparse.

In an earlier study, we investigated the changes in pulmonary function in 34 patients with breast cancer during the administration of adjuvant dose-dense chemotherapy (DDC) [5]. We observed a significant absolute mean reduction of 16.4% in carbon monoxide diffusing capacity (DLCO), with more than half the patients (58.8%) showing a relative decrease of more than 20% from baseline. However, most of the pulmonary injury remained subclinical, and only 5 patients (14.7%) reported grade 1 dyspnea during treatment. To our knowledge, this was the first report on pulmonary function injury with the current widely used standard-of-care DDC protocol [10, 11]. As the patients were otherwise healthy and their disease was potentially curable, long-term follow-up information on their DLCO values is highly important.

The aim of the present study was to investigate the long-term pulmonary and oncological outcomes of these patients as well as the impact of patient and treatment characteristics on DLCO recovery.

## 2. Patients and Methods

The original study cohort consisted of 34 consecutive female patients with breast cancer receiving adjuvant DDC at a single tertiary medical center from September 2006 to April 2007 [5]. None of the patients had any known preexisting lung disease, and all had normal findings on chest X-ray film or computed tomography scan prior to therapy. The original chemotherapy protocol consisted of 4 cycles of IV doxorubicin 60 mg/m^2^ and cyclophosphamide 600 mg/m^2^ (AC) with growth factor support every 14 days, followed by 12 doses of weekly IV paclitaxel 80 mg/m^2^(T). Pulmonary function tests (PFTs) were originally performed before DDC administration (P1), after AC (P2), and after T (P3); 4 patients did not undergo the final P3 PFT. The full DDC regimen was completed in 33 patients. The remaining patient received 3 additional cycles of AC with 5-fluorouracil instead of T; she showed no reduction in DLCO on the P2 PFT.

For the present study, all original PFTs were reviewed and reanalyzed. Pulmonary follow-up data were available for 27 patients of the original cohort who underwent another PFT (P4) after a median of 27 months from onset of DDC (range, 8–97). We excluded 2 patients from the analysis because their later PFT was performed after disease recurrence. The primary endpoint of the study was the DLCO value at P4. The secondary endpoints were symptoms, additional therapies, disease recurrence, and mortality; these data were obtained from the medical records.

### 2.1. Pulmonary Function Tests

All PFTs, at baseline and thereafter, were performed at the same pulmonary laboratory using the Medical Graphics Pulmonary Function System (1070-series 2, St. Paul, MN, USA). They included spirometry, lung volume, and DLCO by single-breath technique. The predicted values of the parameters were obtained from the regression equations of the European Community for Coal and Steel [12]. DLCO values were corrected for hemoglobin level and recorded as a percentage of the predicted value according to height and age at the time of testing, thereby correcting for the effects of body structure and aging. Studies have shown that owing to the high variability among patients, DLCO changes over time and interventions are better analyzed as a percentage of the baseline DLCO values [13]. In the present report, we focused on the effect of a decline in DLCO during any part of the DDC regimen on later outcomes. Therefore, the maximal DLCO reduction in the original study was recalculated continuously as the greater reduction from baseline at either P2 or P3. As intersession variability in healthy adults has been reported be as high as 9% [12] and stricter reports state that an intersession variation of at least 16% is needed to be deemed significant [14], patients were considered to have a DDC-related DLCO injury (DRDI) if their maximal DLCO reduction was more than 20%. This cutoff was also in line with the median 19.9% reduction in DLCO seen from P1 to P3. DLCO recovery in the follow-up cohort was defined continuously as the difference in percentage from baseline between the DLCO value at P4 and the lower DLCO value at either P2 or P3.

### 2.2. Statistical Analysis

We used individual and means graphs to show the differences that occurred in DLCO over time and between subgroups. A longitudinal general linear model with complementary descriptive statistics was employed to evaluate differences over time and between groups. In order to evaluate associations between DLCO recovery and time of follow-up, patient characteristics, and additional therapies, we used the chi-square (*χ*^2^) test for categorical parameters and *t*-tests or Pearson correlation for continuous parameters, as appropriate. Associations between DRDI and oncological outcomes were evaluated with the Cox proportional hazard regression model.

## 3. Results

The patients' clinical and treatment characteristics are displayed in Table 1. No significant differences were observed in any of the parameters between the original and the follow-up cohorts. Specifically, the rate of DRDI was similar in the two cohorts (58.8% vs. 64%, *p*=0.31).

**Figure 1:**
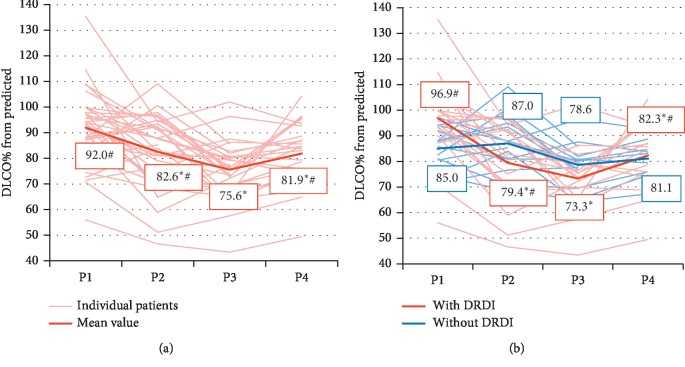
(a) Individual and mean DLCO values measured at 4 time points. A longitudinal general linear model shows a significant effect of time as both a linear and a quadratic covariate (*F*(1, 87) = 14.68, *p* < 0.001 and *F*(1, 87) = 10.26, *p*=0.002, respectively), indicating significant changes in DLCO and in its trend, i.e., a reduction during DDC until P3 and partial recovery at follow-up. (b) When patients are grouped by the presence of DRDI, the longitudinal general linear model yields a significant interaction between DRDI and both time covariates (*F*(1, 85) = 7.96, *p*=0.006 and *F*(1, 85) = 6.79, *p*=0.011, respectively). Therefore, in patients with DRDI, the decrease to P3 and the recovery at P4 were significant, whereas in patients without DRDI, the changes were not significant. Thin pale lines represent individual patients; thick bright lines represent mean values. ^*∗*^Significantly lower than P1 at *p* < 0.05. ^#^Significantly higher than P3 at *p* < 0.05. Abbreviations: DLCO, carbon monoxide diffusing capacity; DRDI, dose-dense chemotherapy-related DLCO injury; P1, prior to DDC administration; P2, after AC; P3, after T; P4, later follow-up.

The changes in DLCO values over time were analyzed using a longitudinal general linear model with time as a continuous variable. In this manner, we were able to analyze time as both a linear and quadratic covariate. The effect of both was found to be significant (*F*(1, 87) = 14.68, *p* < 0.001 and *F*(1, 87) = 10.26, *p*=0.002, respectively). The significant quadratic effect of time indicates a significant change in the trend of DLCO, which was negative up to P3 and positive thereafter (Figure 1(a)). Complementary descriptive statistics supported this finding, showing a significant reduction in DLCO from P1 to P3 (92.0% vs. 75.6%, *p* < 0.001) and a significant recovery at P4 (75.6% vs. 81.9%, *p*=0.002). Additionally, mean DLCO at P4 was significantly lower than mean DLCO at P1 (81.9% vs. 92.0%, *p*=0.003).

In order to identify differences between patients with and without DRDI, a similar longitudinal general linear model was employed to explore the effects of presence of DRDI and time as a linear and quadratic covariate, with an interaction term between DRDI and both time covariates (Figure 1(b)). The results showed a significant effect of DRDI (*F*(1, 32) = 8.43, *p*=0.007) and a significant interaction between DRDI and both time covariates (*F*(1, 85) = 7.96, *p*=0.006 and *F*(1, 85) = 6.79, *p*=0.011, respectively). On further exploration of this interaction, we found that the effect of time on DLCO and its trend was significant in patients with DRDI (*F*(1, 52) = 14.06, *p* < 0.001) but not in patients without DRDI (*F*(1, 33) = 0, *p*=0.98). Complementary descriptive statistics showed that patients with DRDI had both a significant decrease at P3 (96.9% vs. 73.3%, *p* < 0.001) and a significant recovery at P4 (73.3% vs. 82.3%, *p*=0.013), although the value at P4 was still significantly lower than that at P1 (82.3% vs. 96.9%, *p*=0.001). Interestingly, the baseline DLCO was higher for patients who had DRDI than for patients who did not (96.9% vs. 85.0%, *p*=0.015). Pearson correlation analysis between the maximal DLCO reduction and the baseline DLCO value yielded significant results (*r* = −0.506, *p*=0.002).

**Figure 2:**
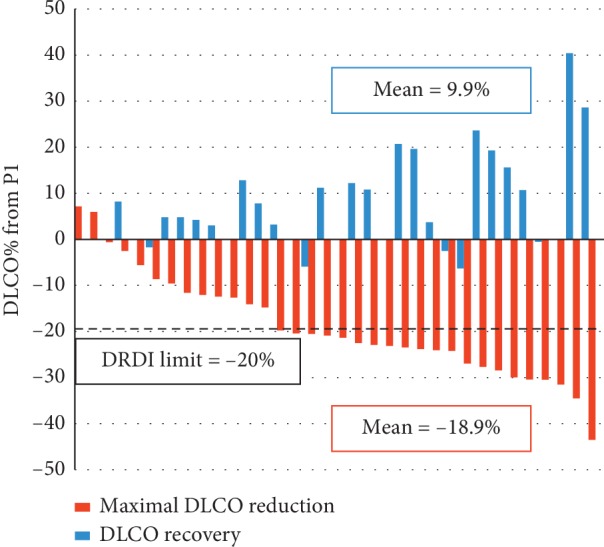
Maximal DLCO reduction during DDC and DLCO recovery at later follow-up. Abbreviations: DLCO, carbon monoxide diffusing capacity; DRDI, dose-dense chemotherapy-related DLCO injury.

The mean maximal DLCO reduction during DDC was 18.9%, and the mean DLCO recovery was 9.9% (Figure 2). Nevertheless, a reduction of more than 20% from baseline was still present on the follow-up PFT in 5 of the 25 patients (20%).

**Figure 3:**
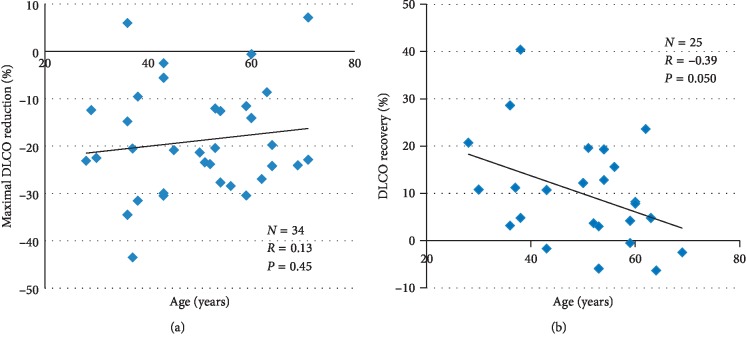
Maximal DCLO reduction (a) and recovery (b) according to age at dose-dense chemotherapy onset. Abbreviations: DLCO, carbon monoxide diffusing capacity.

To evaluate the association between age and both maximal DLCO reduction and DLCO recovery, we used the Pearson correlation. As reported in the original study, we found that age was not associated with maximal DLCO reduction (*r* = 0.13, *p*=0.45; Figure 3(a)), but it was inversely correlated with DLCO recovery (*r* = −0.39, *p*=0.05; Figure 3(b)).

**Figure 4:**
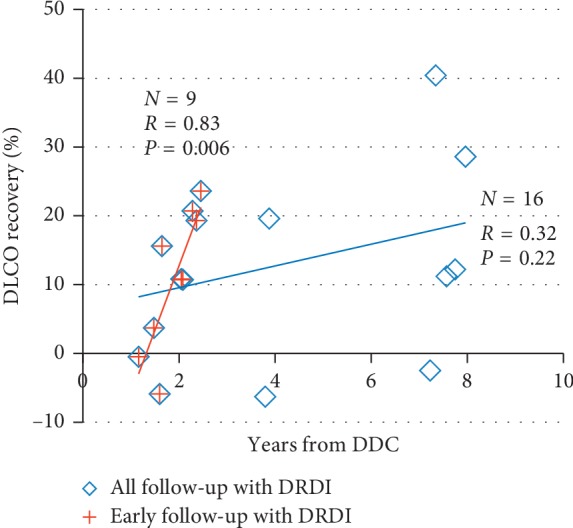
DLCO recovery according to the length of time passed from DDC onset to the follow-up PFT in patients with DRDI. Abbreviations: DDC, dose-dense chemotherapy; DLCO, carbon monoxide diffusing capacity; DRDI, DDC-related DLCO injury.

No significant correlation was found between DLCO recovery, and the length of time elapsed between DDC administration and P4 (*r* = 0.14, *p*=0.42). However, analysis of the results recorded in the initial 3 years after DDC revealed a trend-level correlation (*r* = 0.49, *p*=0.06). On separate analysis of the patients with DRDI (Figure 4), the correlation strengthened and became significant (*r* = 0.83, *p*=0.006).

No additional significant associations were found between DLCO recovery and patient and treatment characteristics, including body mass index, smoking status, staging, endocrine therapy, trastuzumab treatment, and breast or chest wall tangential irradiation, with or without lymph node fields. When only the patients with DRDI were analyzed, we found that the mean DLCO value in recovery was lower in smokers than in nonsmokers (4.5% and 15.3%) and in patients who were treated with trastuzumab than in patients who were not (6.5% and 14.0%). However, neither of these differences was statistically significant (*p*=0.16 and *p*=0.25, respectively).

After a median follow-up of 11 years, none of the patients had received a diagnosis of chronic pulmonary disease. Five of the 25 patients (20%) reported nonspecific dyspnea and/or fatigue (while not under endocrine therapy) one year or more after DDC administration. These patients had significantly lower DLCO values at P4 relative to baseline than patients without such complaints (80.5% vs. 92.1%, *p*=0.02). The complaints were not age-related.

**Figure 5:**
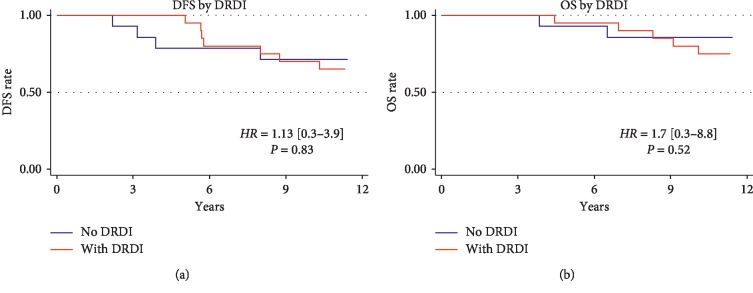
Disease-free survival and overall survival in patients with and without DRDI. Abbreviations: DRDI, dose-dense chemotherapy-related carbon monoxide diffusing capacity injury; DFS, disease-free survival; OS, overall survival.

Regarding oncological outcomes, the disease recurred in 11 of the original cohort of 34 patients (32.4%), of whom 7 died (20.6%). The 20 patients in the original cohort with DRDI (58.8%) appeared to be at higher risk for both these events, but the association did not reach statistical significance (Figure 5). After the follow-up PFT, second malignancies developed in 3 patients (pancreatic adenocarcinoma, cholangiocarcinoma, and uterine cancer).

## 4. Discussion

The present study investigated pulmonary function over time in patients with breast cancer receiving adjuvant DDC. To our knowledge, this is the only study that has investigated sequential PFTs with the AC-T regimen. Several other studies reported pulmonary follow-up of comparable length. In two long-term studies of PFT after breast irradiation, the results were contradictory. Erven et al. [15] and Jaén et al. [16] showed milder reductions in DLCO during radiotherapy than in our cohort (4–7.5%). Only 35% and 51% of their patients, respectively, received adjuvant chemotherapy, almost exclusively based on cyclophosphamide, methotrexate, and 5-fluorouracil. Erven et al. [15] reported a significant mean maximal decline of 9% in DLCO at 10 years compared to baseline whereas Jaén et al. [16] reported a mean maximal decline of 3% from baseline at 2 years after treatment with a relative recovery at 7 years. These differences may be explained by the fact that the radiation therapy in the cohort of Erven et al. [15] was delivered without computed tomography planning and with more extensive lymph node fields, practices that have been found to increase lung exposure to radiation [17].

The radiotherapy administered to the cohort of Jaén et al. [16] might be more in accordance to that used in our cohort, and its mild effect on DLCO might explain the absence of an association between DLCO recovery and radiotherapy parameters in the present study. In addition, the 2-year nadir in DLCO and the later recovery in the earlier study may be in line with the trend we observed of DLCO recovery in the first 3 years after DDC administration.

Bhalla et al. [6] reported a significant 12.6% mean reduction in DLCO in 150 patients with high-risk locally advanced breast cancer treated with cyclophosphamide/doxorubicin/5-fluorouracil (CAF) as induction therapy prior to either standard-dose chemotherapy with cyclophosphamide, cisplatin, and bischloroethylnitrosourea or high-dose chemotherapy with autologous bone marrow transplantation. Follow-up data beyond 2 years were available for 19 (25%) and 22 (29%) patients from each group, respectively. The DCLO values of the standard-dose group did not decline further during treatment, with a final mean decrease of 10% from the pre-CAF baseline. The high-dose group had a mean DLCO reduction of up to 60% from baseline during treatment, and most of the patients were treated with prednisone. After 2 years, the reduction averaged 22% from the pre-CAF value. The DLCO injury associated with the CAF induction protocol was further explored by Bhalla et al. using bronchoalveolar lavage analysis. The results showed an increase in alveolar cellular inflammation markers such as IL-6, IL-8, neutrophils, and lymphocytes compared to healthy volunteers [6]. This effect is thought to be attributable mainly to the cyclophosphamide, whose by-product acrolein interferes with the antioxidant system of tissues, resulting in oxidative stress, apoptosis, and necrosis. This toxicity can rarely lead to interstitial pneumonitis and fibrosis [18, 19]. If oxidative stress is the driving mechanism of pulmonary injury, these findings might also explain the presence of DRDI in the patients with a higher baseline DLCO in our cohort. Since tissue hypoxia is associated with cancer treatment resistance [20, 21], it might be associated with lesser toxicity as well.

The greater DLCO injury in our original study (18.9%) compared to the CAF induction protocol might be explained by the greater dose intensity of AC, which was delivered every 2 weeks instead of every 3 weeks. The prophylactic use of growth factor support might also have contributed to the DLCO reduction, as it has been known to exacerbate chemotherapy-related pulmonary toxicity through activation of neutrophils and a proinflammatory cytokine response [22]. In addition, our patients received paclitaxel, which may cause pulmonary toxicity such as pulmonary infiltrates and hypersensitivity reactions and, in rare cases, pneumonitis [23, 24]. The mechanism for hypersensitivity is thought to be non-IgE-mediated given that most reactions occur during the first and second doses [25]. However, the contribution of paclitaxel to the DLCO injury, which worsened after its administration, is unclear, since it was given with proper premedication and none of the patients acquired clinical symptoms of pneumonitis.

The mean DLCO recovery observed during follow-up in the present cohort (9.9%) was roughly half the mean maximal DLCO reduction (18.9%) observed in the original study; nevertheless, in 5 patients (20%), there was a reduction of more than 20% from their baseline DLCO. Although our patients apparently had a more pronounced recovery than the cohort of Bhalla et al. [6], their patients were more heavily treated afterwards, so it is hard to draw any clear conclusions from this comparison.

Among the various symptoms affecting breast cancer survivors, fatigue was reported to have the strongest impact on global quality of life [4]. Researchers have suggested that the fatigue might be partly explained by dyspnea due to a mild, lasting pulmonary injury from either chemotherapy, radiotherapy, or both [2, 5]. The association we found between DLCO injury and the reported dyspnea and fatigue on later clinical follow-up supports this explanation.

Older age was found to be significantly related to lesser DLCO recovery. Age was not associated with DLCO injury or recovery in our first report or in any of the others reviewed [5–9, 15, 16]. Therefore, this finding might be due to the more substantial DLCO injury seen in our original study, which made the effect of age on recovery more pronounced, achieving statistical significance. Other studies found that older survivors had poorer HRQOL [2, 26, 27]. As suggested earlier, their lower DLCO recovery may have been a contributory factor.

While aging in general is known to cause a decline in DLCO in healthy adults [28, 29], its impact is seen over decades rather than a few years of follow-up, as in our cohort of mostly middle-aged women. In addition, since all DLCO measurements were performed in the same pulmonary laboratory and compared to the predicted age-specific values, we believe this effect is related more to the treatment than to aging until the last follow-up PFT.

The effects of trastuzumab on DLCO recovery and of DRDI on oncological outcomes did not reach statistical significance, but they are still intriguing and warrant further investigation.

### 4.1. Strengths and Limitations

We had full information for all patients, and no patient was lost to follow-up. Each patient served as her own control; therefore, the size of our cohort was sufficient for measuring changes over time and interventions. However, the modest size of the cohort limited our ability to perform subgroup analyses and discern effects of various patient and treatment characteristics on pulmonary outcomes. In addition, the follow-up PFTs were performed over a prolonged range of time, making it difficult to quantify the correlation between time until follow-up and pulmonary recovery.

## 5. Conclusion

DLCO injury after DDC administration is observed in almost all patients with breast cancer. Most patients recover and return to their near-baseline pulmonary function. However, some have a lasting symptomatic DLCO injury, with older patients at higher risk. The significant DLCO injury observed with standard-of-care therapy in patients with potentially curable breast cancer is evident even years afterwards and might play a role in the decrease in overall health and quality of life of survivors. Effects of other breast cancer therapeutics on pulmonary function need to be better elucidated. Practitioners should be aware of these long-term effects of treatment.

## Figures and Tables

**Table 1:** Patient and treatment characteristics: original and follow-up cohorts.

Characteristic	Original cohort (*n* = 34)	Follow-up cohort (*n* = 25)	*p* value
Age at diagnosis (yr), mean ± std	49.6 ± 12.5	49.9 ± 11.5	0.92
Body mass index (kg/m^2^), mean ± std	27.0 ± 5.2	26.7 ± 5.0	0.82
Smoker	6 (17.6)	5 (20.0)	0.55
Stage			
I	4 (11.8)	4 (16.0)	0.2
II	21 (61.8)	14 (56.0)	0.24
III	9 (26.5)	7 (28.0)	0.73
Adjuvant endocrine therapy	24 (70.6)	19 (76.0)	0.24
Adjuvant trastuzumab	7 (20.6)	5 (20.0)	0.89
Triple negative breast cancer	8 (23.6)	5 (20.0)	0.41
Radiotherapy			
Tangential lymph nodes	32 (94.1)	23 (92.0)	0.38
Tangential + regional lymph node fields	15 (44.1)	12 (48.0)	0.44
DDC-related DLCO injury	20 (58.8)	16 (64.0)	0.31
Breast cancer recurrence	11 (32.4)	6 (24.0)	0.08
Breast cancer-related death	7 (20.6)	4 (16.0)	0.27
Values are *n* (%) unless otherwise stated.			
Abbreviations: DDC, dose-dense chemotherapy; DLCO, carbon monoxide diffusing capacity			

## Data Availability

The clinical data used to support the findings of this study are included within the article.
